# HIV related hypochlorhydria does not appear to respond to anti-retroviral therapy in Zambian adults: a case control study

**DOI:** 10.11604/pamj.2018.31.128.11850

**Published:** 2018-10-22

**Authors:** Violet Kayamba, Aaron Shibemba, Kanekwa Zyambo, Douglas Corbett Heimburger, Douglas Morgan, Paul Kelly

**Affiliations:** 1 Tropical Gastroenterology & Nutrition Group, Department of Internal Medicine, University of Zambia School of Medicine, Nationalist Road, Lusaka, Zambia; 2 Cancer Diseases Hospital, Pathology section, Nationalist Road, Lusaka, Zambia; 3 Vanderbilt Institute of Global Health, Vanderbilt Medical Center, 2525 West End Avenue, Suite 750, Nashville, 37203, Tennessee, USA; 4 Blizard Institute, Barts & The London School of Medicine and Dentistry, Queen Mary University of London, 4 Newark Street, London E1 2AT, UK

**Keywords:** HIV, intestinal metaplasia, hypochlorhydria, gastric atrophy, pepsinogen

## Abstract

**Introduction:**

Human Immunodeficiency Virus (HIV) infection is associated with hypochlorhydria but the mechanism is unknown. The objective of this study was to determine effects of anti-retroviral therapy (ART) on gastric physiology as measured by validated markers.

**Methods:**

We studied HIV infected individuals who were either ART-naïve or on treatment with undetectable viral loads. We measured H.pylori IgG antibodies, pepsinogen (PG) 1 and 2 levels and fasting gastrin-17 using Biohit GastroPanel®. Gastric antral biopsies and juice were obtained for histology and pH respectively. Also included were historical data from HIV negative participants (n = 72) in a previous study, for reference.

**Results:**

We enrolled 84 HIV positive individuals with a median age 42 years (IQR 37-40 years). 55(66%) were female, 32(38%) were ART naïve, and 52(62%) were on ART. Hypochlorhydria (pH>4) was present in 48(57%) of the HIV positive and 18(25%) of the HIV negative individuals (OR 4: 95% CI 1.9-8.5, P<0.001) with no significant effect of ART (OR 0.9: 95% CI 0.3-2.3, P = 0.82). Hypochlorhydria was not associated with the serological detection of corpus atrophy using low PG 1:2 ratio (OR 2.1: 95% CI 0.5-10.2, P = 0.37) or GastroPanel^®^ algorithm, (OR 0.7: 95% CI 0.01-60.1, P = 1.0). ART reduced the frequency of low PG 1:2 ratio (P = 0.001), but not the histological detection in the antrum of atrophy or non-atrophic gastritis.

**Conclusion:**

ART use is associated with reduced serological evidence of corpus atrophy but has no effect on fasting pH, supporting earlier data that suggest that the mechanism of HIV-associated hypochlorhydria is multifactorial.

## Introduction

Sub-Saharan Africa bears over two-thirds of the global Human Immunodeficiency Virus (HIV) burden. The prevalence of HIV in Zambia is 12.9% with 63% of these individuals being on antiretroviral therapy (ART) [1]. The use of ART has resulted in HIV infected individuals living longer and healthier lives and the classical opportunistic infections becoming less common [2]. The profile of health conditions in general and in HIV infection is beginning to change with a notable shift towards non-communicable diseases [2]. The gut plays an important role in HIV infection and it has been established that gut-associated lymphoid tissue continues to harbour HIV even when there is complete serological viral load suppression [3, 4]. Hypochlorhydria (gastric pH greater than 4) is twice as common in HIV infection probably due to the presence of the virus [5, 6]. Hypochlorhydria is typically a consequence of the loss of gastric parietal cell mass resulting from atrophy, a known risk factor for gastric cancer. There is no evidence to date of parietal cell loss in HIV infection [5]. In addition, the long-term consequences of persistent hypochlorhydria in HIV infection, including gastric cancer are unknown [7]. It is however known that hypochlorhydria predisposes to intestinal infections [5], micronutrient deficiencies [8] and other health outcomes. Over 15 years ago ART was rolled out to the general public in sub-Saharan Africa. Many HIV infected persons have benefited from this therapy, whose influence on gastric physiology remains uninvestigated. For the evaluation of gastric histology, endoscopy with biopsies is the gold standard but it is invasive and costly [9]. The option of serological assessment, "serological biopsy2 is therefore attractive. The GastroPanel^®^ (Biohit Helsinki, Finland) is a non-invasive diagnostic test for gastric pathology including four-biomarkers, pepsinogen 1 (PG 1), pepsinogen 2 (PG 2), *Helicobacter pylori* IgG (*H.pylori* IgG) antibodies and Gastrin-17. The utility of the GastroPanel^®^ may prove to be a useful tool in detection of atrophic gastritis and gastric cancer risk assessment [10, 11]. However, some studies have questioned its usefulness [12, 13]. The aim of this study was to compare these markers of gastric physiology in HIV infected ART-naïve individuals and those who have taken treatment long enough to completely suppress HIV in blood. In addition, we included data from HIV negative individuals as a point of reference. To our knowledge, this tool has not been applied to the problem of hypochlorhydria in HIV infected individuals.

## Methods

**Participant enrollment:** We enrolled participants from the University Teaching Hospital (UTH) in Lusaka, Zambia between October 2014 and July 2015. This was a case control study which included asymptomatic HIV infected individuals who were either ART-naïve as cases or on ART with full viral suppression ("ART-treated") as controls. Only individuals above the age of 18 years and who had given written consent were included in the study. Excluded were those with evidence of ART failure, use of gastric acid suppressing medication, presence of active gastrointestinal disease or significant co-morbidities such as chronic liver or kidney disease. For comparison, all proven HIV negative community volunteers from a previous study were included [14]. The university of Zambia biomedical research ethics committee (Ref no. 004-04-14) and the national health research authority approved this study.

**Upper gastrointestinal endoscopy:** Using a Pentax EG-2990i instrument, we conducted upper gastrointestinal endoscopies on HIV positive participants after an overnight fast. A full assessment of the upper gastrointestinal tract was carried out first according to a standard protocol. After flushing the biopsy channel, gastric juice was aspirated for pH determination, using pH paper test strips (Sigma Chemical Company St Louis, USA), which measure pH to the nearest 0.5. Hypochlorhydria was defined as gastric pH>4.0. Four gastric biopsies were obtained from the gastric antrum (three of which were from the region of the incisura) and immediately placed in formalin and sent to the histopathology laboratory for analysis. The biopsies were stained with hematoxylin and eosin and Giemsa special staining for *H.pylori*. The pathologist (AS) characterized gastric histology by considering infiltration of the lamina propria by acute or chronic inflammatory cells to diagnose active or chronic non-atrophic gastritis (NAG). The presence of gastritis with increased distance between the individual glands (glandular loss) and the condensation of reticulin fibers in the lamina propria was used to diagnose chronic atrophic gastritis (CAG) [15]. The complete Operative Link on Gastritis Assessment (OLGA) could not be employed as no corpus biopsies were obtained.

**Laboratory analysis:** Using the Biohit GastroPanel^®^ ELISA kits, (Biohit Helsinki, Finland), serum samples were analysed for pepsinogens 1 and 2, gastrin-17 and *H.pylori* IgG antibodies. A PG 1:2 ratio of less than 3.0 was considered low, indicative of gastric body atrophy. To designate a result as low PG 1 or gastrin-17, we used the cut-offs 30 μg/L and 1 μg/L respectively. Samples with enzyme immunounits greater than or equal to 30 were reported to be positive for the presence of *H.pylori* IgG antibodies. Using the GastroPanel^®^ algorithm [13] and GastroSoft^®^ analysis panel available online (Gastrosoft^®^) [16], we were able to designate each participant as having corpus or antral atrophy, multifocal atrophy, non-atrophic gastritis or normal mucosa. The GastroPanel^®^ algorithm utilizes results for H.pylori, PG 1 and gastrin-17, while in the Gastrosoft^®^, PG 2 is included as well. To measure the HIV viral load in plasma, we used the COBAS AmpliPrep/COBAS TaqMan HIV-1 Test, version 2 (Roche Molecular Systems, Branchburg, USA).

**Statistical analysis and sample size calculation:** We used previously published data on HIV positive patients with hypochlorhydria. In that study, 25% of the HIV negative individuals had hypochlorhydria while the proportion among those with HIV infection (with a high CD4) count was 50%, giving an odds ratio of 4 (P<0.001) [5]. Using these proportions at a power of 80%, 66 patients would be needed in each group. In included in this study were 84 HIV positive and 72 HIV negative individuals. Statistical analysis was done in STATA 13 (College Station, TX, USA). Medians and interquartile ranges were obtained for continuous variables while proportions were employed for categorical variables. The Fisher's exact test was used to compare binary variables and presented as odds ratios with 95% confidence intervals. For the comparison of continuous variables, Kruskal-Wallis test and the Spearman rank's correlation were used. In all instances, a two-sided P value of <0.05 was considered statistically significant.

## Results

We enrolled 84 clinically asymptomatic HIV positive participants. 32(38%) were ART-naïve and 52(62%) had been on ART for 2-14 years (median 10 years). The median age was 43 years (IQR 37-40 years); 66% were female. All individuals in the ART-treated group had undetectable viral loads and significantly higher CD4 counts than the ART-naïve group (medians 522 cells/μL and 297 cells/μL respectively, P=0.001). Our comparison group included 72 clinically asymptomatic HIV negative participants from a previous study (Table 1).

**Table 1:** Demographic and biological characteristics of the three groups: HIV positive and ART naive, HIV positive and ART-treated, and HIV negative

	HIV positive, ART naïve n=32	HIV positive, ART treated n=52	HIV negative n=72	P
Age in years, median (IQR)	36(30-44)	45(40-52)	41(30-53)	0.001
Female, n(%)	20(63)	35(67)	39(54)	0.35
BMI, (median IQR)	22(20-23)	23(21-27)	21(20-25)	0.08
Gastric pH, Median (IQR)	4.8(2.0-6.0)	5.5(2.0-6.0)	1.5(1.0-4.4)	<0.001
Hypochlorhydria, n(%)	19(59)	29(56)	18(25)	<0.001
PG 1, Median (IQR)	73.1(55.8-110)	69.7(45.2-104.1)	80.7(53.2-107.3)	0.15
*Low PG 1, μg/L, n(%)	3(10)	4(8)	2(3)	0.21
PG 2, μg/L Median (IQR)	14.7(9.8-38.6)	12.7(6.9-20.7)	14.0(9.9-22.0)	0.25
PG 1:2 ratio, Median (IQR)	4.3(2.2-9.1)	5.4(4.2-6.9)	5.9(4.3-7.7)	0.36
**Low PG 1:2 ratio, n(%)	10(37)	4(7.6)	6(8)	0.001
Gastrin-17 μg/L, Median (IQR)	10.4(4.9-14.4)	6.0(3.6-14.7)	***	0.08
Low gastrin-17, n(%)	1(3)	5(10)	***	0.40
H.pylori IgG antibody EIU, Median (IQR)	61.4(38.2-86)	76.4(38.2-99.1)	***	0.12
H.pylori positive	26(81)	41(79)	***	0.10

* Using 30 μg/L as the cut-off

** Using 3.0 as the cut-off

**** In the previous study, gastrin-17 and H.pylori IgG antibodies were not measured

**Figure 1:**
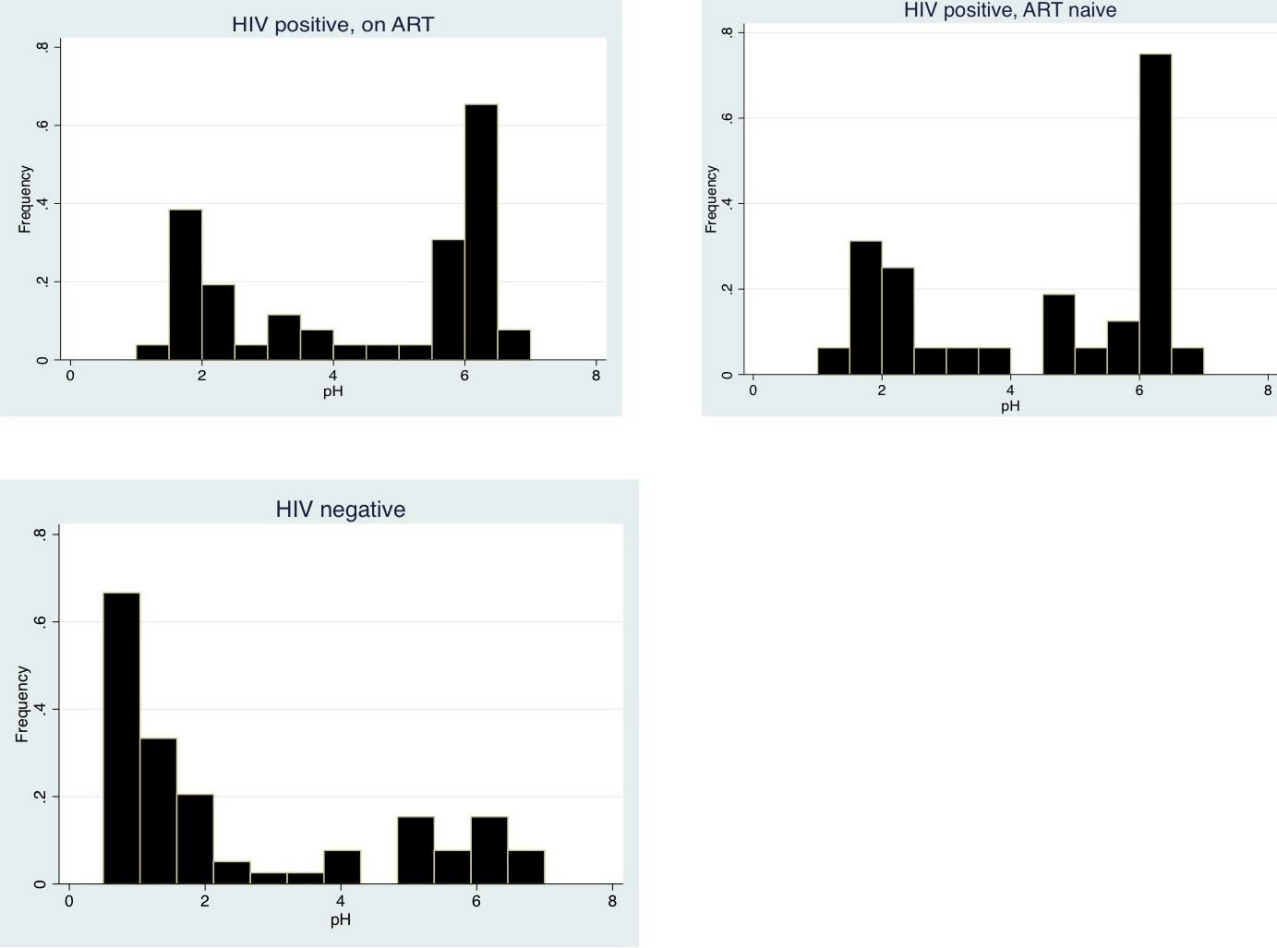
Gastric pH observed in three groups; HIV positive and ART naive, HIV positive and ART-treated and HIV negative

**Fasting gastric pH:** Gastric pH was bimodally distributed in all three groups, with two peaks of frequency between 1 to 3 and 5 to 6 (Figure 1). Hypochlorhydria was present in 48(57%) of the HIV positive and 18(25%) of the HIV negative individuals (OR 4.0: 95% CI 1.9-8.5, P<0.001) with no significant difference between the ART-naïve and ART-treated groups (OR 0.9: 95% CI 0.3-2.3, P = 0.82). The median age among those with hypochlorhydria was significantly higher, 44(36-53) years versus 40(30-49) years, P = 0.02. Hypochlorhydria was associated with the presence of *H.pylori* IgG antibodies in the ART-treated group (OR 8.6: 95% CI 1.4-89.3, P = 0.007) but not in the ART-naïve group (OR 1.6: 95% CI 0.2-14.2, P = 0.67). In addition, there was no link between hypochlorhydria and BMI (P = 0.13) or CD4 count (P = 0.50) (data not shown in tables).

**Pepsinogen 1:2 ratio:** The median values of PG 1, PG 2 and PG 1/PG 2 ratio was not significantly different in the three groups (Table 1). However, 37% of ART-naïve group had a low PG 1:2 ratio while in both the ART-treated and HIV negative groups the proportion was around 8% (Table 1). Using an unsupervised stepwise logistic regression, we found that being HIV positive increased the odds of having a low PG 1:2 ratio. However, individuals who were female, on ART and less than 40 years had significantly lower odds of having a low PG 1:2 ratio (Table 2).

**Table 2:** Factors associated with pepsinogen 1:2 ratio of less than 3.0; data for all

	Low PG 1:2 ratio, n=20	Normal PG 1:2 ratio, n=131	Univariable		Multivariable, n=125	
			OR(95% CI)	P	OR(95% CI)	P
Female	8(40)	85(65)	0.4(0.1-1.0)	0.047	0.15(0.03-0.65)	0.01
HIV positive	14(70)	65(50)	2.1(0.8-8.0)	0.099	24(4.0-154)	0.001
On ART	4(20)	48(37)	0.4(0.1-1.5)	0.207	0.02(0.0-0.24)	0.001
Age less than 40 years	7(35)	61(47)	0.6(0.2-1.8)	0.470	0.1(0.02-0.73)	0.02

the three groups included, n=151

**GastroPanel^®^ algorithm:** Analysis using the GastroPanel^®^ algorithm indicated that 44(52%) of HIV positive individuals had NAG. Antral atrophy was detected in 19(23%), corpus atrophy in 2(2%) and multifocal atrophy in 4(5%). There was no significant difference in the occurrence of these lesions in the ART-naïve and treated groups. The proportion of NAG on histology was 72(95%) with that of antral atrophy being 6(8%). All individuals with NAG detected using the GastroPanel^®^ also had it on histology, with a positive predictive value (PPV) of 100%, sensitivity of 71% and 100% specificity. The PPV for GastroPanel^®^ detection of antral atrophy was 26%, with a sensitivity of 50% and a specificity of 80%.

**GastroSoft^®^ online software:** Using this alternative online software, 50(80%) of all the HIV positive individuals were positive for *H.pylori* infection, 2(3%) had antral atrophy and 10(13%) had corpus atrophy. Similarly, there was no difference between the ART-naïve and treated groups (data not shown). The PPV of this test for antral atrophy was 75%, with a sensitivity of 25% and a specificity of 97%.

**Serological levels of pepsinogens in comparison with histological diagnoses of the antrum in HIV infection:** individuals with histological diagnosis of antral atrophy were significantly older (median 55, IQR 52-59 years) than those without (median 41, IQR 37-49 years; P=0.009). HIV positive individuals with non-atrophic gastritis had increased levels of both PG 1 and 2. However, those with atrophic gastritis reduced levels, although the difference was not statistically significant (Table 3).

**Table 3:** Serological levels of pepsinogens in individuals with atrophic gastritis, non-atrophic gastritis and H.pylori infection diagnosed on histology

Histological diagnosis		PG 1(μg/L) Median (IQR)	PG 2(μg/L) Median (IQR)	PG 1:2 ratio Median (IQR)	Fasting gastrin-17 Median (IQR)
Atrophic gastritis	Present	44.9(37.2-114)	8.6(4.5-10.7)	5.8(3.6-7.5)	16.9(5.9-22.1)
	Absent	73.1(50.1-102)	14.3(8.5-22.5)	5.5(4.0-7.3)	6.6(4.1-13.6)
P value		0.23	0.24	0.84	0.11
Non-atrophic gastritis	Present	74.6(50.9-110)	14.7(9.1-21.9)	5.3(3.6-7.0)	7(4.3-14.7)
	Absent	39.7(30.4-43.6)	5.3(3.9-6.3)	8.4(5.5-10.3)	4.5(1.9-11.8)
P value		0.008	0.007	0.19	0.34
H.pylori seen	Present	86.4(60.3-114.2)	17.1(8.7-21.3)	5.4(4.6-6.6)	7.4(4.7-15.2)
	Absent	43.5(30.2-59.5)	6.2(4.5-9.8)	7.8(6.1-9.3)	2.9(0.9-10.8)
P value		0.02	0.48	0.42	0.02

## Discussion

In this study, we have shown that HIV-associated hypochlorhydria [5, 17] is similar between ART-naïve and ART-treated individuals but not any of the serological indicators of corpus atrophy [18]. In contrast, ART reduced the occurrence of low PG 1:2 ratio, a reported serological indicator of gastric corpus atrophy. This apparently paradoxical finding suggests that hypochlorhydria in HIV infection is not a consequence of gastric atrophy. The pathogenesis of hypochlorhydria in HIV infection remains obscure. In 1988, Lake-Bakaar et al. suggested that it could be due to the presence of parietal cell autoantibodies [17]. Their conclusions were however based on a very small number of patients and there has been no further studies supporting this theory. Another explanation could be that persistent gastric inflammation drives the low acid production despite the lower prevalence of *H.pylori* in HIV infection [19]. Our results showed an association between hypochlorhydria and *H.pylori* in the ART-treated group but not the ART-naïve group. This could be a suggestion of *H.pylori* re-colonization with ART use without any effect on hypochlorhydria. Quantification of parietal cell mass with direct measure of maximal gastric acid output in HIV infection is one possible approach to understanding HIV associated hypochlorhydria. Transcriptomic or proteomic analysis of gastric mucosal tissue may also be useful. Histological detection of gastric premalignant lesions such as atrophy is not without sampling error. To increase sensitivity, we obtained four antral biopsies using ordinary white light endoscopy. We were able to establish that the occurrence of gastric atrophy was not affected by ART use. We then evaluated the influence of ART on PG 1, PG 2 and gastrin-17 compared to histological diagnoses. PG 1 is produced in the corpus and its levels are reduced in corpus atrophy. When the corpus is inflamed without glandular loss, levels of PG 1 in blood increase [20]. PG 2 is produced in the corpus, antrum and duodenum. Similar to PG 1 its levels will increase in non-atrophic gastritis [20]. In this study, we report consistent trends showing a significant increase in both PG 1 and 2 with non-atrophic gastritis and a decrease of both markers in atrophy. Similarly, Kitamura et al concluded that serum pepsinogen analysis could be used to predict the presence of *H.pylori* gastritis [21].

However, on histology only antral atrophy was analysed. We were therefore not surprised that that the decrease of pepsinogens in antral atrophy was insignificant. The third serological marker evaluated in this study was fasting gastrin-17. The G cells of the antrum produce basal gastrin-17 [20]. It is expected that levels of gastrin-17 would decrease when the antral mucosa is atrophic. Paradoxically our results did not show any association between antral atrophy and gastric-17. In addition, we did not find any effect of HIV disease progression on the levels of fasting gastrin-17 (which would be indicative of antral inflammation) as determined by some other investigators. The levels of serum pepsinogens were similar regardless of the presence of *H.pylori* on histology. Histology is specific for *H.pylori* yet its sensitivity is hampered by the patchy colonization of the stomach [22]. The median age of participants in the ART-naïve group was significantly lower than in the ART-treated or the HIV negative groups. As the incidence of gastric atrophy increases with advancing age, we would expect the occurrence of low PG 1:2 ratio to be less in the ART-naïve group. However, our results showed a significantly higher proportion of low PG 1:2 ratio in the younger ART-naïve group, an effect that seem to be reversed on ART. We therefore concluded that HIV infection could be directly or indirectly influencing the production of pepsinogens, through a mechanism similar to that causing hypochlorhydria. Several investigators have published reports supporting the use of "serological biopsy" to evaluate gastric physiology, but this has not yet been examined in HIV infection. It is clear that HIV is associated with physiological changes and therefore any such investigative strategies would warrant specific validation. We therefore explored the utility of the GastroPanel^®^ kit for detection of gastric atrophy in HIV infection. To analyse our data, we used both the algorithm for GastroPanel and the online software GastroSoft^®^. Results from both analyses were very similar, with both showing no significant difference between the ART-naïve and ART-treated groups. All the individuals designated as having NAG using the GastroPanel^®^ also had it on histology, giving the test a very good specificity. However, the sensitivity was much lower as the GastroPanel^®^ missed some cases of NAG. From our findings, the GastroPanel^®^ would not be helpful for detecting antral atrophy in this population. We were unable to fully assess its usefulness for corpus atrophy, as there were no histology results available. This is the first study to examine the influence of ART on gastric physiology. The notable limitation of this study was the lack of gastric corpus biopsies. Secondly, measuring the maximal gastric acid output as done by Welage et al. would have allowed us to better understand hypochlorhydria in HIV infection [23]. Conversely, this study draws its strength from the fact that the analysis was done in healthy HIV infected volunteers with HIV negative controls.

## Conclusion

We have shown that ART does not reverse HIV associated hypochlorhydria. The mechanism of low acid output in HIV infection is yet to be established.

### What is known about this topic

- HIV infection is associated with hypochlorhydria;
- Anti-retroviral therapy can suppress the viral load to undetectable levels;
- Use of the GastroPanel^®^ has not been validated in HIV infection.

### What this study adds

- Anti-retroviral therapy does not reverse HIV associated hypochlorhydria;
- The occurrence of gastric atrophy is not affected by anti-retroviral therapy;
- Anti-retroviral therapy reduces the occurrence of low pepsinogen 1:2 ratio.

## Competing interests

The authors declare no competing interests.
